# Exploring the endangerment mechanisms of *Hipposideros pomona* based on molecular phylogeographic methods

**DOI:** 10.1002/ece3.10653

**Published:** 2023-10-20

**Authors:** Wei Liu, Yan Hao, Xinhang Song, Liqun Ma, Jing Li, Jingying He, Yanzhen Bu, Hongxing Niu

**Affiliations:** ^1^ College of Life Sciences Henan Normal University Xinxiang China

**Keywords:** conservation, gene flow, genetic diversity, geographical isolation, quaternary climate change

## Abstract

The endangerment mechanisms of various species are a focus of studies on biodiversity and conservation biology. *Hipposideros pomona* is an endangered species, but the reasons behind its endangerment remain unclear. We investigated the endangerment mechanisms of *H. pomona* using mitochondrial DNA, nuclear DNA, and microsatellite loci markers. The results showed that the nucleotide diversity of mitochondria DNA and heterozygosity of microsatellite markers were high (*π* = 0.04615, *H*_O_ = 0.7115), whereas the nucleotide diversity of the nuclear genes was low (*THY*: *π* = 0.00508, *SORBS2*: *π* = 0.00677, *ACOX2*: *π* = 0.00462, *COPS7A*: *π* = 0.00679). The phylogenetic tree and median‐joining network based on mitochondrial DNA sequences clustered the species into three clades, namely North Vietnam‐Fujian, Myanmar‐West Yunnan, and Laos‐Hainan clades. However, joint analysis of nuclear genes did not exhibit clustering. Analysis of molecular variance revealed a strong population genetic structure; IMa2 analysis did not reveal significant gene flow between all groups (*p* > .05), and isolation‐by‐distance analysis revealed a significant positive correlation between genetic and geographic distances (*p* < .05). The mismatch distribution analysis, neutral test, and Bayesian skyline plots revealed that the *H. pomona* population were relatively stable and exhibited a contraction trend. The results implied that *H. pomona* exhibits female philopatry and male‐biased dispersal. The Hengduan Mountains could have acted as a geographical barrier for gene flow between the North Vietnam‐Fujian clade and the Myanmar‐West Yunnan clade, whereas the Qiongzhou Strait may have limited interaction between the Hainan populations and other clades. The warm climate during the second interglacial Quaternary period (*c*. 0.33 Mya) could have been responsible for species differentiation, whereas the cold climate during the late Quaternary last glacial maximum (*c*. 10 ka BP) might have caused the overall contraction of species. The lack of significant gene flow in nuclear microsatellite loci markers among the different populations investigated reflects recent habitat fragmentation due to anthropogenic activities; thus, on‐site conservation of the species and restoration of gene flow corridors among populations need immediate implementation.

## Graphical abstract

This study was based on the mitochondrial DNA, nuclear DNA, and microsatellite markers, using molecular phylogeographic methods to explore the mechanism of endangerment. Results suggested that the lack of significantly gene flow in nuclear microsatellite loci markers among the different populations investigated reflects recent habitat fragmentation due to anthropogenic activities; thus, on‐site conservation measures should be implemented to promote effective protection of the species.

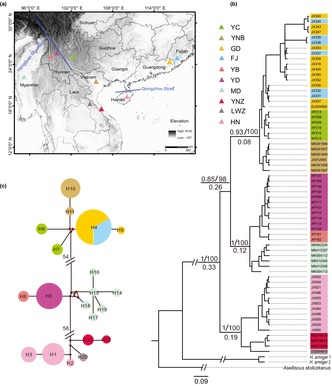

## 1 INTRODUCTION

Biodiversity is the basis of human survival and development, and it is an ecologically important parameter (Pimm et al., 2014). However, with the rapidly increasing global population and anthropogenic activities, biodiversity is sharply declining, and many species have either become extinct or are on the verge of extinction (Amano et al., 2021; Ceballos et al., 2020; Frankham, 1995). Recent studies in the field of biodiversity and conservation biology are focusing on the mechanisms of species endangerment (Mi et al., 2023). The following key issues should be elucidated for the protection of endangered species: habitat requirements of endangered species; process of endangerment; causes of endangerment; and the trends and possibilities of species extinction (Payne et al., 2016; Pulliam & Babbitt, 1997).

Species endangerment is usually the result of interactions between multiple factors. Extensive research has been conducted on species endangerment from different perspectives, such as genetics, population ecology, physiological ecology, and community ecology (Blanco et al., 2021; Menges, 2000; Robertson et al., 2019; Schwartz et al., 2007). The mechanisms of species endangerment mainly include genetic depletion, secondary extinction, habitat destruction and fragmentation, changes in the physical and chemical environment of habitats, introduction of few species and biological invasion, and overhunting driven by economic interests (Cardoso et al., 1998; Kuussaari et al., 2009; Lin & Wang, 2002). Genetic factors play key roles in species endangerment, but they are often overlooked and should be investigated in detail (Frankham, 1995).

Studies involving molecular phylogeography elucidate the population genetic status, genetic structure, gene flow levels, and geographic distribution patterns of a species (Avise et al., 1987; Avise & Walker, 1998), thus providing a scientific basis, such as threatened status, endangerment mechanisms, and the delimitation of conservation management units, for developing conservation strategies (Emerson et al., 2001; Schwartz et al., 2007). The geographical distribution pattern of a species is the result of the interaction between multiple factors, such as paleoclimatic changes, topography, and population history (Lin et al., 2014; Seeholzer & Brumfield, 2018). The Pleistocene period faced the greatest environmental changes in Earth's history, thus climate and geological changes in this period had a profound impact on the genetic diversity patterns of species (Avise & Walker, 1998; Hofreiter et al., 2004). However, the harsh environment during the ice age led to a rapid decline in the populations of many species, posing a risk of genetic resource depletion (Song et al., 2020). Evaluating the impact of surface morphology and paleoclimatic changes on the phylogeographic structure of animals is essential for understanding the patterns of population geographical structures and species conservation (Dussex et al., 2014; Newton et al., 1999).

Andersen's Roundleaf bat (*Hipposideros pomona*) is widely distributed throughout South and Southeast Asia, including southern China, Vietnam, Laos, Myanmar, Cambodia, Malaysia, India, Bangladesh, and Thailand, etc. (Bates & Harrison, 1997; Simmons, 2005), and is recognized as an endangered species by the IUCN (Srinivasulu et al., 2020). Currently, the classification status, distribution range, habitat environment, echolocation, mother‐infant acoustic recognition, and morphological changes of *H. pomona* are being investigated (Bounsavane et al., 2010; Jin et al., 2015; Murray et al., 2012); however, the endangerment mechanisms of this species have not been elucidated.

In this study, we analyzed *H. pomona* mitochondrial DNA, nuclear DNA, and microsatellite markers (nuclear simple sequence repeats; nSSRs) using molecular phylogeographic methods to investigate (1) its genetic diversity and phylogenetic relationships to infer the dispersal route through divergence time estimation; (2) the impact of surface topography on gene flow among populations through population genetic structure and gene flow analysis; and (3) the population history dynamics and the impact of glacial climate during the Pleistocene period on population history dynamics. We aimed to evaluate the endangerment mechanisms of *H. pomona* and provide reference data for its protection by evaluating the impact of surface topography and ancient climate change on the phylogeographic structure of this species.

## 2 MATERIALS AND METHODS

### 2.1 Sample collection and DNA extraction

**FIGURE 1:**
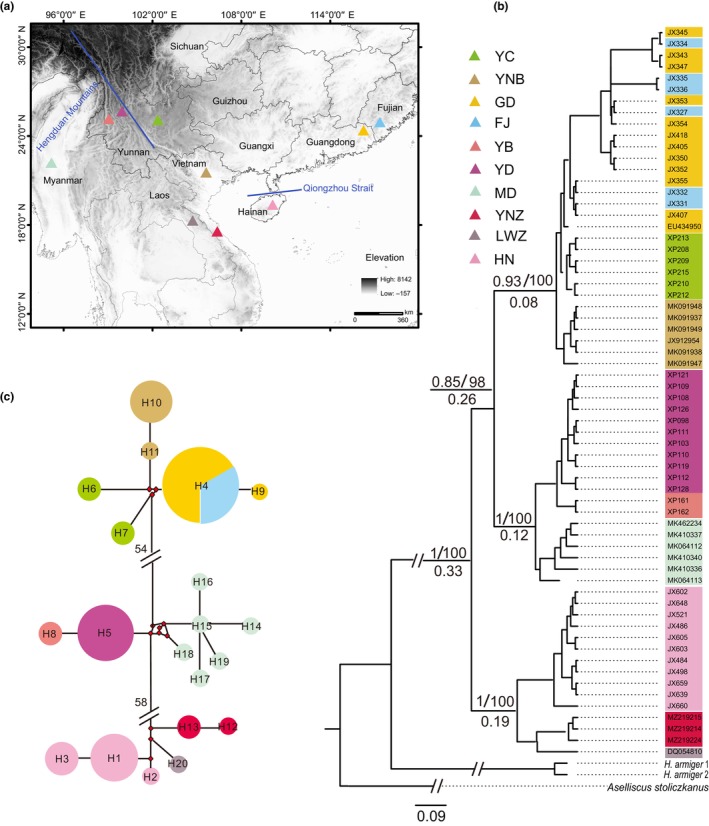
Sampling locations of *Hipposideros pomona*, median‐joining network and ultrametric tree based on *Cytb* sequences. (a) Collection sites for this survey. FJ, Fujian; GD, Guangdong; HN, Hainan; LWZ, Central Laos; MD, Myanmar; YB, Yunnanbaoshan; YC, Yunnanchuxiong; YD, Yunnandali; YNB, North Vietnam; YNZ, central Vietnam; (b) Bayesian ultrametric tree inferred based on the mitochondrial *Cytb* sequences. Values above the branches indicate the Bayesian posterior probabilities and maximum likelihood bootstrap support values; numbers below branches are the times to the most recent common ancestor (in Ma); (c) Median‐joining network constructed using mitochondrial haplotypes. Each circle corresponds to a haplotype whose size is proportional to its total frequency, and the color of the circles represents geographical location. The numbers in the network represent mutational steps between haplotypes.

In this study, we obtained 47 *H. pomona* individuals from six habitats in the Yunnan, Guangdong, Fujian, and Hainan provinces of China (Figure 1a, Table S1). According to Hill (1963), *H. pomona* is found in various countries, including China, Vietnam, Laos, and Myanmar. However, there remains controversy regarding the appropriate name for the species found in China, specifically whether it should be named as *H. pomona* or *H. gentilis* (Hill, 1963; Srinivasulu & Srinivasulu, 2018; Wei et al., 2021). The wing membrane tissue of each animal was sampled with a punch, and the animals were released in situ; the tissue samples were stored in 95% ethanol. DNA from the tissue samples was extracted using an Ezup Column Animal Genomic DNA Purification Kit (Sangon Biotech). The concentration and purity of the DNA were confirmed using a UV spectrophotometer, and the DNA samples were diluted to a concentration of 100 ng/μL and stored at −20°C until further use. Moreover, we downloaded 10 cytochrome *b* gene (*Cytb*) sequences of *H. pomona* from China, Myanmar, Vietnam, and Laos from the GenBank database for data analysis (accession numbers: EU434950, MK064112, MK064113, MK462234, MK410336, MK430029, MZ219215, MZ219224, JX912954, and DQ054810). We abbreviated Fujian, Guangdong, Hainan, Central Laos, Myanmar, Yunnanbaoshan, Yunnanchuxiong, Yunnandali, North Vietnam, and Central Vietnam as FJ, GD, HN, LWZ, MD, YB, YC, YD, YNB, and YNZ, respectively. All fieldwork was conducted in accordance with the Law of the People's Republic of China on the Protection of Wildlife. All experiments involving live bats followed the guidelines of the American Society of Mammalogists (Sikes et al., 2011).

### 2.2 Polymerase chain reaction and microsatellite genotyping

We amplified one mitochondrial gene, namely *Cytb*, four nuclear gene fragments (*THY*, *SORBS2*, *ACOX2*, *COPS7A*), and eight microsatellite loci as molecular markers from the isolated DNA samples using polymerase chain reaction (PCR). The PCR products were sequenced using a 3730XL sequencer (ABI). The primers used for this experiment were designed as per previously published studies (Eick et al., 2005; Irwin et al., 1991; Yusefovich et al., 2020; see Table S2). Eight microsatellite loci (Liu et al., 2008) were genotyped in 47 *H. pomona* DNA samples (Table S3).

### 2.3 Genetic diversity, phylogenetic, and divergence time analyses

The sequencing data were spliced and manually modified using Sequencer 5.4 (Gene Codes Corporation). Multiple sequence alignments were performed using the MEGA 5 (Tamura et al., 2011) software.

Haplotype diversity (*h*) and nucleotide diversity (*π*) of *Cytb*, *THY*, *SORBS2*, *ACOX2*, and *COPS7A* were calculated using the DNASP v6 (Rozas et al., 2017) software; since the LWZ population contained only one individual, it was combined with the YNZ population (geographically closer) to calculate genetic diversity. For microsatellite data analysis, observed heterozygosity (*H*_O_) and expected heterozygosity (*H*_E_) were calculated using the POPGENE 1.32 (Nei, 1972) software; and Hardy–Weinberg equilibrium (HWE), linkage disequilibrium, and null allele frequencies for each locus were tested using the Genepop (Rousset, 2008) software. We used the Wilcoxon method in R 4.1.2 (R Core Team, 2021) to determine the difference between *H*_O_ and *H*_E_.

We selected *Hipposideros armiger* and *Aselliscus stoliczkanus* as outgroups and used the maximum likelihood (ML) and Bayesian inference (BI) methods to construct phylogenetic of *Cytb* sequences. The GTR + F + G4 substitution model was selected based on the Akaike Information Criterion in ModelFinder (Kalyaanamoorthy et al., 2017). Nodal support for the ML trees was calculated using 1000 bootstrap replicates in MEGA 5. In MIRBANES 3.2.6 (Huelsenbeck & Ronquist, 2001), four Markov chains were run for 200 million generations and sampled every 100 generations. The first 25% of the resulting trees were discarded as burn‐in.

Laboratory sequencing data were used for the joint analysis of nuclear gene (*THY*, *SORBS2*, *ACOX2*, *COPS7A*) sequences, and *H. armiger* was used as an outgroup. ML phylogenies were inferred using the MEGA 5 software under the K2 + G + I model for 1000 bootstraps. BI analysis was performed using MRBAYES 3.2.6, and four Markov chains were run for 400 million generations based on the optimal model.

The time to the most recent common ancestor (TMRCA) for the major lineages was estimated in BEAST 1.10.4 (Suchard et al., 2018), using the GTR + F + G4 substitution model. A *Cytb* mutation rate of 0.013 substitutions site^−1^ Myr^−1^ (Lin et al., 2014; Thong et al., 2012) was used as the fixed mean substitution rate in the analysis. Under a strict molecular clock model, Markov chain MCMC runs were conducted thrice for 20 million generations, with sampling every 1000 generations. The results were combined after a 10% burn‐in using a logcombiner in the BEAST package. The convergence of the MCMC chains was examined by estimating an effective sampling size (ESS) > 1000 using Tracer 1.71 (Rambaut et al., 2018). The final maximum clade credibility (MCC) tree was prepared using the TreeAnnotator in the BEAST package.

### 2.4 Population genetic structure and gene flow

We use Arlequin version 3.5 (Excoffier & Lischer, 2010) to calculate the pairwise Φ_ST_ and *F*_ST_ values based on *Cytb* sequences and microsatellites markers, respectively, to analyze genetic differentiation among the populations. First, to examine the population genetic structure, the sources of variation within and among populations were assessed by performing analysis of molecular variance analysis (AMOVA) of *Cytb* sequences and microsatellite data using Arlequin 3.5. This AMOVA produces estimates of variance components and *F*‐statistic analogs, designated here as phi‐statistics, reflecting the correlation of haplotypic diversity at different levels of hierarchical subdivision (Excoffier et al., 1992). Second, a median‐joining network of *Cytb* haplotypes and nuclear genes was constructed using the Network10 (Bandelt et al., 1999) software to determine the relationships among the haplotypes. Finally, the STRUCTURE (Pritchard et al., 2000) was used to elucidate the population genetic structure of the microsatellite data, and the results were analyzed using the online tool STRUCTURE HARVESTER (https://taylor0.biology.ucla.edu/struct_harvest/) to determine the most likely *K*‐value for the population.

Using the *Cytb* sequences and microsatellite data, we analyzed the correlation between genetic and geographical distances. We conducted Mantel tests using IBDWS 3.23 (Jensen et al., 2005) to evaluate whether the distribution of genetic variation conforms to the isolation‐by‐distance (IBD) model. Finally, we analyzed the *Cytb* and microsatellite data using the IMa2 (Hey, 2010) algorithm to estimate gene flow among populations. The program utilizes the isolation with migration model to analyze genetic data obtained from a pair of loosely related populations or species. The result is an estimate of the joint‐posterior probability density function for the model parameters. To obtain more reliable computational results, a large prior distribution range was first set for all the parameters to be estimated, and a small number of sampling iterations were performed. After verifying the ESS and distribution trend for each parameter, reasonable adjustments were made to the prior distribution ranges of the parameters. The final running parameters included 20 chains with 2 million MCMC runs, in which gene genealogy was recorded every 100 runs. The recommended upper limits of parameters for population size, migration rate, and differentiation time in the operation manual (Hey, 2011), were 10, 1, and 10, respectively.

### 2.5 Population dynamics

Neutral tests and mismatch distribution analyses were performed in Arlequin 3.5, and Tajima's *D* (Tajima, 1989) and Fu's *F*s (Fu, 1997) tests were sensitive to bottleneck effects or population expansion, thus we determined whether the populations experienced expansion. Population dynamics was evaluated by constructing Bayesian Skyline Plot (BSP) using BEAST 1.10.4 under the following conditions; strict molecular clock model, mutation rate of 0.013 Myr^−1^, MCMC runs for 20 million generations, and samples recording every 1000 generations.

## 3 RESULTS

### 3.1 Genetic diversity, phylogenetic, and divergence time analysis

Genetic diversity analysis of *H. pomona Cytb* sequence revealed that the overall *π* was 0.04615, whereas the *π* of the nuclear genes *THY*, *ACOX2*, *COPS7A*, and *SORBS2* was 0.00667, 0.00642, 0.00679, and 0.00677, respectively. However, the nuclear gene loci exhibited low *π*, except for the YC population, and the overall *π* indicated fixed distinct sequences in each population. For the eight microsatellite loci, except for loci P5 (where only one allele was detected in the YC and YB populations), we could not determine whether the alleles deviated from the HWE or whether null alleles were present. Consistent signs of deviation from the HWE or linkage disequilibrium were not detected for the remaining loci (*p* > .05). The overall *H*_E_ and *H*_O_ of *H. pomona* were 0.7425 and 0.7115, respectively, with no significant difference between both values (Wilcoxon *W* = 31.0, *p* = .240). The overall *H*_O_ was slightly lower than the *H*_E_, and heterozygote deficiency was also observed (Table 1).

**TABLE 1:** Genetic diversity analyses: sample size (*n*), nucleotide diversity (*π*), expected heterozygosity (*H*_E_), and observed heterozygosity (*H*_O_) for *Hipposideros pomona*.

Population		HN	FJ	GD	YD	YC	YB	MD^a^	YNB^a^	LWZ^a^	Total
*Cytb*	*n*	11	6	12	11	6	2	6	6	4	64
	*π*	0.00093	0.00000	0.00015	0.00000	0.00234	0.00000	0.00760	0.00029	0.01360	0.04615
*THY*	*n*	11	5	10	11	6	2	—	—	—	45
	*π*	0.00000	0.00000	0.00123	0.00037	0.01448	0.00000	—	—	—	0.00667
*SORBS2*	*n*	11	5	10	9	4	1	—	—	—	40
	*π*	0.00844	0.00000	0.00035	0.00186	0.01787	—	—	—	—	0.00677
*ACOX2*	*n*	10	4	9	10	4	1	—	—	—	38
	*π*	0.00000	0.00564	0.00686	0.00040	0.01992	—	—	—	—	0.00642
*COPS7A*	*n*	11	5	10	10	5	2	—	—	—	43
	*π*	0.00769	0.00113	0.00126	0.00236	0.00453	0.00141	—	—	—	0.00679
nSSR		11	6	11	11	6	2	—	—	—	47
	*H*_E_	0.7073	0.7652	0.7291	0.7273	0.7348	0.7917	—	—	—	0.7425
	*H*_O_	0.6591	0.7292	0.6682	0.6920	0.7083	0.8125	—	—	—	0.7115

^a^ *Cytb* sequence downloaded from the GenBank, which were not used in microsatellite analyses.

**FIGURE 2:**
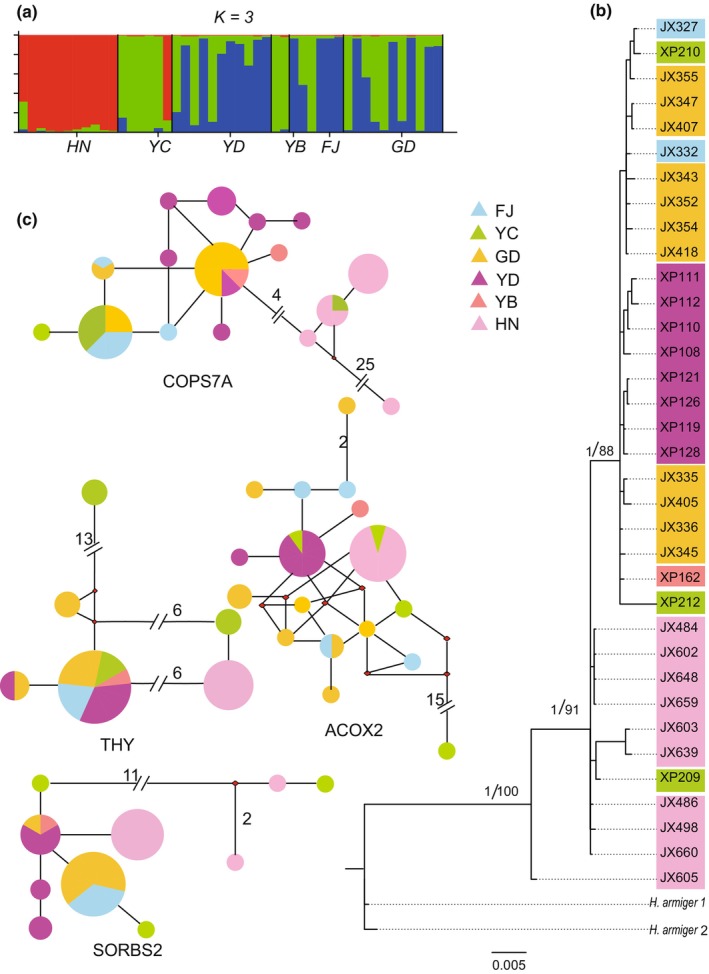
Median‐joining network and Bayesian phylogenetic tree based on nuclear gene sequences, Bayesian clustering results from STRUCTURE based on microsatellite date of *Hipposideros pomona*. (a) Bayesian clustering results (*K* = 3); Population abbreviations are identical to those included in Figure 1; (b) Bayesian phylogenetic tree constructed using a combination of four nuclear genes. Values above the branches indicate the Bayesian posterior probabilities and maximum likelihood bootstrap support values; (c) median‐joining network composed of nuclear genes. Each circle corresponds to a haplotype whose size is proportional to its total frequency, and the color of the circles represents the geographical location. The numbers in the network represent mutational steps between haplotypes.

All restored ML and BI topologies were similar and divided into three main branches. Clade compositions were as follows: North Vietnam‐Fujian clade (YNB, GD, FJ, and YC populations), Myanmar‐West Yunnan clade (MD, YB, and YD populations), Laos‐Hainan clade (LWZ, YNZ, and HN populations) (Figure 1b). The phylogenetic tree based on *THY*, *SORBS2*, *ACOX2*, and *COPS7A* sequences differed from those constructed using *Cytb* sequences, and they did not possess clear geographical population branches (Figure 2b). The MRCA of all examined *H. pomona* individuals could be traced back to 0.33 Mya (95% highest posterior density [HPD]: 0.04–2.8 Mya). The MRCA of the North Vietnam‐Fujian and Myanmar‐West Yunnan clades was dated to 0.26 Mya (95% HPD: 0.03–2.14 Mya). The MRCA of the Laos‐Hainan clade was dated to 0.19 Mya (95% HPD: 0.02–1.38 Mya) (Figure 1b).

### 3.2 Population genetic structure

*Cytb* exhibited high and significant overall genetic differentiation, with Φ_ST_ values (except for the GD and FJ) ranging from 0.6 to 1 (*p* < .05). Moreover, based on the nSSR dataset, low and significant *F*_ST_ values were observed, with the *F*_ST_ values of paired populations ranging from 0.04376 to 0.21735 (*p* < .05) (Table 2).

**TABLE 2:** Genetic differentiation coefficient of *Cytb* (lower triangle) and microsatellites (upper triangle).

	HN	FJ	GD	YD	YC	YB	YNB	LWZ	MD
HN		0.18884*	0.20379*	0.21735*	0.15292*	0.19794*	—	—	—
FJ	0.99205*		0.04376*	0.11345*	0.10744*	0.15186	—	—	—
GD	0.99333*	−0.06883		0.08648*	0.05847*	0.12495*	—	—	—
YD	0.99390*	1.00000*	0.99862*		0.10325*	0.07961*	—	—	—
YC	0.98233*	0.60000*	0.69008*	0.98625*		0.05860*	—	—	—
YB	0.98906*	1.00000*	0.99765*	1.00000*	0.96635*		—	—	—
YNB	0.99120*	0.97872*	0.97228*	0.99829*	0.83636*	0.99585*	—	—	—
LWZ	0.90039*	0.93000*	0.95752*	0.95060*	0.91192*	0.84942*	0.92950*	—	—
MD	0.95838*	0.93333*	0.95580*	0.84853*	0.91542*	0.69079	0.93267*	0.85087*	—

* *p* < .05.

AMOVA revealed significant intergroup, intragroup, and intrapopulation differences between mitochondria DNA and microsatellites markers (*p* < .05), with the highest percentage of variation between groups (81.28%) observed for mitochondria DNA, and the highest percentage of variation within populations (84.31%) observed for microsatellites markers (Table 3).

**TABLE 3:** AMOVA of *Hipposideros pomona Cytb* and microsatellites markers. The three different groups were North Vietnam‐Fujian clade (YNB, GD, FJ, and YC populations), Myanmar‐West Yunnan clade (MD, YB, and YD populations) and Laos‐Hainan clade (LWZ, YNZ, and HN populations).

Source of variation	Sum of squares	Percentage of variation	Fixation indices
*Cytb*			
Among groups	1363.15	81.28	*F*_CT_: 0.81 (*p* < .01)
Among populations within groups	253.34	15.90	*F*_SC_: 0.85 (*p* < .01)
Within populations	58.61	2.81	*F*_ST_: 0.97 (*p* < .01)
nSSR			
Among groups	33.34	9.53	*F*_CT_: 0.10 (*p* < .05)
Among populations within groups	16.23	6.15	*F*_SC_: 0.07 (*p* < .01)
Within populations	253.82	84.31	*F*_ST_: 0.16 (*p* < .01)

Median‐joining network analysis of *Cytb* sequences elucidated three major haplotype clades with strong geographical structure, which were consistent with the structure of the phylogenetic tree. No haplotype was shared by the geographically isolated groups except for the GD and FJ populations (Figure 1c). However, the network of nuclear genes differed from the *Cytb* network, with shared haplotypes among different geographic populations (Figure 2c). Structure analysis was performed using the Δ*K* method, where *K* = 3 corresponded to the most likely number of genetic clusters (Figure 2a).

IBD analysis revealed that the genetic distance based on *Cytb* sequences and microsatellites was significantly correlated with geographical distance (*p* < .05). This indicated that gene flow occurred between the populations but was limited by distance. IMa2 analysis did not reveal any significant gene flow between populations. We observed one‐way insignificant gene flow from North Vietnam‐Fujian clade to Laos‐Hainan clade (population migration rate 2NM = 1.874, *p* > .05), whereas gene flow was not observed between other clades (2NM < 0.001, *p* > .05).

### 3.3 Population dynamics

**FIGURE 3:**
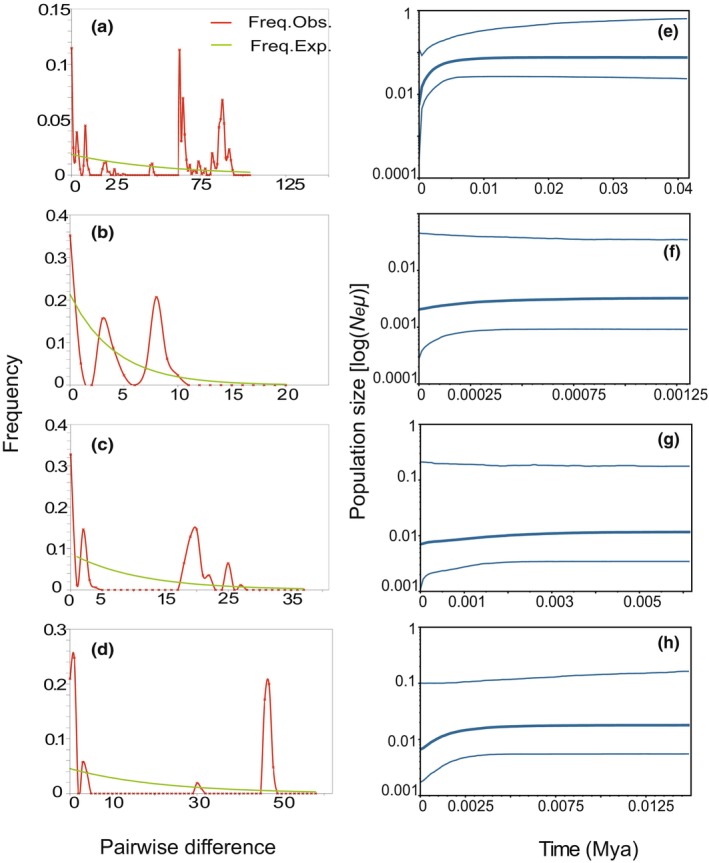
Mismatch distribution and Bayesian Skyline Plot based on *Cytb* sequences. (a) Mismatch distribution based on all *Cytb* sequences; (b) Mismatch distribution based on North Vietnam‐Fujian clade; (c) Mismatch distribution based on Myanmar‐West Yunnan clade; (d) Mismatch distribution based on Laos‐Hainan clade; (e) Skyline plot based on all *Cytb* samples; (f) Skyline plot based on North Vietnam‐Fujian clade; (g) Skyline plot based on Myanmar‐West Yunnan clade; (h) Skyline plot based on Laos‐Hainan clade.

The three clades exhibited multimodal mismatch distribution (Figure 3a–d). The results based on *Cytb* neutrality test elucidated that the Tajima *D*'s and Fu's *F*s in the North Vietnam‐Fujian clade (Tajima *D*'s = 0.15796, Fu's *F*s = 2.59756), Myanmar‐West Yunnan clade (Tajima *D*'s = −0.28915, Fu's *F*s = 3.35074), and Laos‐Hainan clade (Tajima *D*'s = 0.22129, Fu's *F*s = 4.87948) were either non‐significant, negative, or >0. The BSPs did not reveal significant expansion of the species, and the effective population number remained stable and began to shrink at *c*. 10 ka BP (Figure 3e–h).

## 4 DISCUSSION

### 4.1 Genetic diversity and dispersal routes of *H. pomona*

In a population, *π* is an important indicator of genetic diversity (Avise et al., 1987). At the species level, *π* of *H. pomona Cytb* (*π* = 0.04615) was higher than that of *H. armiger* (*π* = 0.012) (Lin et al., 2014) and *Rhinolophus macrotis* (*π* = 0.025) (Liu et al., 2019). However, we observed that *π* of most geographic populations (*π* < 0.005) was lower than that observed at the species level. Higher genetic diversity may reflect the long‐term evolutionary history of a large stable population, as well as the mixing of divergent lineages (Avise, 2000; Frankham, 1996; Grant & Bowen, 1998). The YC population exhibited high genetic diversity in mitochondria DNA, nuclear genes, and microsatellite markers. Changes in species distribution reflect glacial and interglacial variation (Roy et al., 1996; Webb & Bartlein, 1992). During the glacial periods, species either withdrew to low latitude areas, or survived in refugium (Ruedi & Castella, 2003). Therefore, places that exhibit high genetic diversity could be the areas that provided refuge to the species during the Quaternary glacial–interglacial climate cycles. YC exhibited high genetic diversity and could have provided refuge during the glacial periods. YC is located on the eastern edge of the Hengduan Mountains, which provided refuge for a variety of animals and plants during the Quaternary glacial period (Xing & Ree, 2017); *H. pomona* may be one of the beneficiaries. Further investigation of the effects of paleoclimatic changes on species distribution can provide evidence for the inferred refugia hypothesis.

In the North Vietnam‐Fujian clade, *π* sequentially decreased from YC (*π* = 0.00234) to YNB, GD, and FJ populations. In the Myanmar‐West Yunnan clade, the MD population (*π* = 0.00706) had the highest *π*. In the Laos‐Hainan clade, the *π* of the LWZ population (*π* = 0.01360) was significantly higher than that of the HN population. Based on the above analysis and the mitochondrial DNA‐based phylogenetic tree of *H. pomona*, we speculated that the MRCA of the three clades could be dated to the second interglacial Quaternary period (*c*. 0.33 Mya). Warm and humid may provide impetus for species divergence; thus, the warm and humid climatic conditions during this period may have been the driving force for species differentiation. The MRCA of the North Vietnam‐Fujian clade and Myanmar‐West Yunnan clade could be dated to the third Quaternary glacial period (*c*. 0.26 Mya). The diffusion route of the North Vietnam‐Fujian clade may have been from YC to the southeast to YNB, GD and FJ in China. The diffusion route of Myanmar‐West Yunnan clade could have led from northeast through the MD to Yunnan, China. In the Laos‐Hainan clade, the MRCA of the HN and LWZ populations can be dated to the third glacial Quaternary period (*c*. 0.19 Mya), and the dispersal route of this clade could have been from LWZ and YNZ to HN.

### 4.2 Effect of the Hengduan Mountains and Qiongzhou Strait on gene flow between populations

Our results revealed significant differences in the population genetic structures of mitochondrial and nuclear markers (including four nuclear genes and microsatellite data). Genetic differentiation analysis (Table 2) and AMOVA (Table 3) indicated the presence of female philopatry and male‐mediated gene flow in *H. pomona*. The low within‐population genetic variation and high genetic differentiation between the populations for mitochondrial DNA could be explained by female philopatry because mitochondrial DNA is maternally inherited. In contrast, the high within‐population genetic variation and low genetic differentiation between populations for nuclear microsatellite loci may be explained by biparental inheritance. This suggests male‐biased dispersal in *H. pomona*. Our results are consistent with those of other bat species, such as *Myotis myotis* (Castella et al., 2001), *Rhinolophus ferrumequinum* (Jang et al., 2021), and *Epiticus fuscus* (Turmelle et al., 2011), indicating female philopatry and male‐biased dispersal in these bats.

The network and structure analyses also revealed differences between mitochondrial and nuclear genes. The mitochondrial network exhibited the same results as the phylogenetic tree, dividing *H. pomona* into three clades, whereas the nuclear gene network did not have obvious branches. Although the structure was also divided into three lineages (Figure 2a), strict phylogeographic relationships among the six populations were not observed, indicating extensive genetic admixture. This could be because we did not obtain complete sequences of the mitochondrial and nuclear genomes, and the gene trees made from a single gene or a small number of genes may have resulted in differences from the actual situation due to incomplete lineage sorting (Avise et al., 1983; Feng et al., 2022).

Geographic isolation is an important factor in the genetic differentiation of many species (Bradburd et al., 2013) and has been observed in birds (Seeholzer & Brumfield, 2018), reptiles (Rodrigues & Diniz‐Filho, 2017), and bats (Liu et al., 2021). Genetic differentiation of *H. pomona* conformed to the geographical distance isolation model. Populations with more geographical proximity exhibited greater genetic similarity (Wright, 1943). This indicates that the populations gradually accumulated genetic variation during diffusion, leading to genetic differentiation.

The geographical populations of *H. pomona* were influenced by geographical isolation, with limited gene flow and a high degree of genetic differentiation, which might be related to various factors such as topography and climate at the time. Mountains and rivers have played important roles in the adaptive evolution of bats (Flanders et al., 2011; Kuo et al., 2015). *Hipposideros pomona* has a small body and poor flying ability, making it difficult for them to sustain long‐distance flights (Norberg et al., 1997). Therefore, these factors may have resulted in limited expansion and significant spatial genetic structure differentiation in *H. pomona*.

Hengduan Mountains and its remnants in China, such as Diancang Mountain and Ailao Mountain, separated the North Vietnam‐Fujian clade from the Myanmar‐West Yunnan clade. The YD and YB populations were located in the southern extension of the Qinghai‐Tibet Plateau, whereas the YC populations were located in the western part of the Yunnan‐Guizhou Plateau. We speculated that the Hengduan Mountains limited gene flow between the North Vietnam‐Fujian clade and the Myanmar‐West Yunnan clade. Hainan Island is geographically separated from the mainland by the Qiongzhou Strait, with a minimum distance of 19 km. Its current isolation date from the Middle Holocene period was approximately 10,000 years ago (Zhao et al., 2007). Hainan Island and the mainland were connected by a minimum of three land bridges during the Pleistocene epoch (Shi et al., 2006). The weak flight ability (Norberg et al., 1997) of *H. pomona* may have prevented gene exchange with other populations after the disappearance of the land bridge connecting Hainan Island to the mainland. After being separated from the mainland, *H. pomona* on Hainan Island retained some original genes.

Like *H. pomona*, *H. armiger* is also reported to have been affected by the Hengduan Mountains and Qiongzhou Strait barriers (Xu et al., 2010), suggesting that both places may have had an important impact on the population structure of many species. In the present study, IMa2 analysis results did not reveal significant gene flow between the mitochondrial and nuclear genes in the various groups. Because of the obstruction of gene flow, communication between populations is reduced, and the chance of self‐breeding is increased, thus strengthening the genetic decline of the species (Jang et al., 2021), which may be one of the reasons for the endangerment of a species. The gene flow of nuclear genes is an indicator of the negative impact of the contemporary environment, especially habitat fragmentation caused by anthropogenic activities (Shaw et al., 2018); thus, gene flow should be carefully monitored to protect this species. Simultaneously, onsite and communication channel protection should be strengthened. This not only protects the haplotype resources of different populations but also provides a channel for gene communication.

### 4.3 Impact of ice age climate on population history during the Pleistocene epoch

The neutral test did not reach significant negative values and the mismatch distribution did not fit the Poisson distribution, indicating that *H. pomona* did not experience rapid expansion. The BSPs revealed that species contraction occurred during the late Quaternary last glacial maximum (LGM) (*c*. 10 ka BP). Cold climatic conditions might be the main reason for the overall contraction of this species. The harsh environment during this period was not conducive to the survival of *H. pomona*, and the population began to decline. Owing to the natural changes in the environment and the frequent human impact, their habitat has been continuously damaged (Guo et al., 2022), resulting in a decrease in their number (Srinivasulu et al., 2020). Furthermore, *H. pomona* exhibits limited dispersal ability and gene flow between populations. Therefore, it is necessary to develop effective scientific policies to strengthen on‐site protection measures for this endangered species.

Although we used molecular methods to explore the endangerment mechanisms in this study, further investigation is required using genome‐wide analysis and denser sampling. Moreover, specimens should be continuously collected from countries, such as Myanmar, India, and Laos, and international cooperation and exchanges should be strengthened. Macro analysis, involving population surveys and biogeography‐based methods, to analyze the relationship between population distribution and the environment, especially the relationship between the cold and warm climate changes during the Quaternary glacial–interglacial cycle, should be performed to elucidate the phylogenetic relationships and for the protection of this species.

## 5 CONCLUSIONS

Although the mitochondrial genetic diversity was comparatively high at the species level, the *π* values of most geographic populations (*π* < 0.005) were lower. The phylogenetic tree constructed using mitochondrial DNA sequences was divided into North Vietnam‐Fujian, Myanmar‐West Yunnan, and Laos‐Hainan clades. The differentiation of species was influenced by the climatic conditions during the Quaternary ice age. The analysis of mitochondrial genes revealed that genetic variation is primarily found among different clades, whereas the analysis of nuclear genes revealed that genetic variation is primarily found within populations, indicating *H. pomona*'s female philopatry and male‐biased dispersal behavior. We did not observe significant gene flow among the clades. The reduction in mitochondrial and nuclear gene flow may be attributed to geographical isolation resulting from the presence of the Hengduan Mountains and Qiongzhou Strait, as well as habitat destruction caused by contemporary anthropogenic activities. Historically speaking, *H. pomona* did not experience a rapid expansion, but instead displayed a decreasing trend following the LGM during the Quaternary period (*c*. 10 ka BP). On‐site conservation of the species and restoration of gene flow corridors among populations need immediate implementation.

## AUTHOR CONTRIBUTIONS

**Wei Liu:** Conceptualization (equal); investigation (equal); software (equal); writing – original draft (lead). **Yan Hao:** Investigation (equal); software (equal); writing – original draft (equal). **Xinhang Song:** Investigation (equal); software (equal). **Liqun Ma:** Data curation (equal). **Jing Li:** Data curation (equal). **Jingying He:** Data curation (equal). **Yanzhen Bu:** Conceptualization (equal); project administration (equal). **Hongxing Niu:** Conceptualization (equal); funding acquisition (lead); project administration (lead).

## CONFLICT OF INTEREST STATEMENT

The authors declare that they have no conflict of interest.

## Supporting information


Appendix S1

## Data Availability

Supplementary information is available for this paper in Appendix S1, and the GenBank accession number of *H. pomona* haplotypes can be found in Table S2 of Appendix S1.
