# Supplementary material for: Exploring the endangerment mechanisms of Hipposideros pomona based on molecular phylogeographic methods
Source: Ecol Evol. 2023 Oct 20;13(10):e10653. doi: 10.1002/ece3.10653 (PMC10587739; doi:10.1002/ece3.10653)
Supplement: Supplementary file 1 — Appendix S1 [file ECE3-13-e10653-s001.doc]

**SUPPLEMENTARY MATERIALS**

**Table S1** Sample collection sites of *H.pomona*

| Collection sites | Number | Latitude | Longitude | Altitude (m) |
| --- | --- | --- | --- | --- |
| Jiaoling City, Guangdong Province（GD） | 11 | 24°32'N | 116°9'E | 109 |
| Nanjing City, Fujian Province  （FJ） | 6 | 24°36'N | 117°15'E | 89 |
| Tunchang City, Henan Province  （HN） | 11 | 19°13'N | 110°6'E | 152 |
| Dali City, Yunnan Province（YN） | 11 | 25°31'N | 99°44'E | 1514 |
| Baoshang City, Yunnan Province（YN） | 2 | 25°12'N | 99°14'E | 1724 |
| Chuxiong City, Yunnan Province（YN） | 6 | 24°57'N | 102°10'E | 1828 |

**Table S2** Mitochondrial and nuclear genes were used in this study, and the maximum length of the obtained sequences, primer sequences, and GenBank accession numbers of *H. pomona* haplotypes are shown.

| **Gene** | **Aligned**  **Fragment Length** |  | **Primer Sequence**  **(Forward and Reverse)** | **Reference** | **accession numbers** |
| --- | --- | --- | --- | --- | --- |
| ***Cytb*** | 1140 | L14724 | CGAAGCTTGATATGAAAAACCATCGTTAACTGCAGTCATCTCCGGTTTACAAGA C |  | OR519889-OR519896 |
|  |  | H15915 |  |  |  |
| ***THY*** | 488 | THYF | GGGTATGTAGTTCATCTTACTTC  GGCATCCTGGTATTTCTACAGTCTTG | Eick et al., 2005 | OR531428-OR531433 |
|  |  | THYR |  |  |  |
| ***SORBS2*** | 569 | SORBS2_L730a | CCATCGAGTCT-YTGCTGGAGGCCTTCT CTTTTCAGC -CTTAATYTGGTCAC | Yusefovich et al., 2020 | OR577360-OR577369 |
|  |  | SORBS2_R1300a |  |  |  |
| ***ACOX2*** | 502 | ACOX2-F1  ACOX2-R1 | CCTSGGCTCDGAGGAGCAGAT  GGGCTGTGHAYCACAAACTCCT | Igea et al., 2010 | OR557556-OR557572 |
| ***COPS7A*** | 707 | COPS-F1  COPS-F1 | TACAGCATYGGRCGRGACATCCA  TCACYTGCTCCTCRATGCCKGACA | Igea et al., 2010 | OR577344-OR577359 |

**Table S3 Sequence, fluorophore, repeat unit and fragment size of fluorescent primers**

| Loci | Primer sequences | Repeat unit | Fragment size | Fluorescent groups |
| --- | --- | --- | --- | --- |
| PE4 | F:GCATCTTTCAGAACACCCAC | (GT)_24_ | 117～149 | HEX |
|  | R:GAGAACTTATCCTTTGTCCCT |  |  |  |
| P6D12 | F:GGGATGGTTATTCTATTCTGCC | (AC)_9_ | 220～242 | FAM |
|  | R:ATGCGTCTTTAGTGACCTTTGA |  |  |  |
| P541 | F:ACACCCAGACAGCAGTCTC | (CA)_12_ | 120～128 | HEX |
|  | R:TGGAACAGTAGACATTGGG |  |  |  |
| P9 | F:AGAAAAGACCAGGACTCAAAG | (AC)_20_ | 241～275 | FAM |
|  | R:GGTAGAAAGAAACGAAAAGGA |  |  |  |
| BAM09 | F:CGCCTCGACAACTTGTTCC | (AC)_14_ | 252～274 | FAM |
|  | R:TCCGAGTGAATGCCAAGTGT |  |  |  |
| PT5B2 | F:CTGCGTGGGTCCGAGTGAAT | (GT)_17_ | 258～286 | FAM |
|  | R:CCGCCTCGACAACTTGTTCC |  |  |  |
| TT18 | F:GCACGCACATAAACACCCTC | (AC)_16_ | 245～271 | FAM |
|  | R:TTGCCAGCGTGATAAAGACC |  |  |  |
| P6B7 | F:CTCTTCATGCTTCTGGTTTT | (TG)_4_N_10_(TG)_4_N_8_(TG)_5_ | 178～200 | HEX |
|  | R:AGAAGGACAAAGGGAAGTAA |  |  |  |
